# Crosslinked Poly(2-oxazoline)s as “Green” Materials for Electronic Applications

**DOI:** 10.3390/polym8010006

**Published:** 2015-12-30

**Authors:** Martin Fimberger, Ioannis-Alexandros Tsekmes, Roman Kochetov, Johan J. Smit, Frank Wiesbrock

**Affiliations:** 1 Polymer Competence Center Leoben, Rosseggerstrasse 12, Leoben 8700, Austria; martin.fimberger@pccl.at; 2 Institute for Chemistry and Technology of Materials, Graz University of Technology, NAWI Graz, Stremayrgasse 9, Graz 8010, Austria; 3 Department of Electrical Sustainable Energy, Delft University of Technology, Mekelweg 4, 2628 CD Delft, The Netherlands; i.a.tsekmes@tudelft.nl (I.-A.T.); roman.kochetov@ch.abb.com (R.K.); 4 Asea Brown Boveri (ABB) Corporate Research, Segelhofstrasse 1k, 5405 Baden-Daettwil, Switzerland

**Keywords:** poly(2-oxazoline)s, crosslinked polymers, thiol-ene click chemistry, permittivity, loss factor, interfacial polarization, electrical conductivity

## Abstract

Poly(2-nonyl-2-oxazoline)_80_-*stat*-poly(2-dec-9′-enyl-2-oxazoline)_20_ and poly(2-dec-9′-enyl-2-oxazoline)_100_ can be synthesized from the cationic ring-opening polymerization of monomers that can be derived from fatty acids from renewable resources. These (co)poly(2-oxazoline)s can be crosslinked with di- and trifunctional mercapto compounds using the UV-induced thiol-ene reaction. The complex permittivity of the corresponding networks increases with the temperature and decreases with the network density. In a frequency range from 10^−2^ to 10^6^ Hz and at temperatures ranging from −20 to 40 °C, the changes of the real part of the complex permittivity as well as the loss factor can be explained by interfacial polarization within the material. At a temperature of 20 °C and a frequency of 50 Hz, the permittivity of the crosslinked (co)poly(2-oxazoline)s covers a range from 4.29 to 4.97, and the loss factors are in the range from 0.030 to 0.093. The electrical conductivities of these polymer networks span a range from 5 × 10^−12^ to 8 × 10^−9^ S/m, classifying these materials as medium insulators. Notably, the values for the permittivity, loss factor and conductivity of these copoly(2-oxazoline)s are in the same range as for polyamides, and, hence, these copoly(2-oxazoline)-based networks may be referred to as “green” alternatives for polyamides as insulators in electronic applications.

## 1. Introduction

**Scheme 1:**
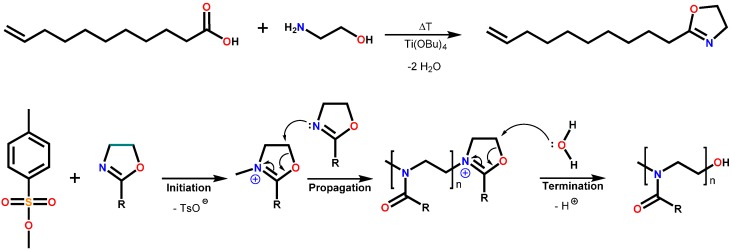
Schematic representations of the synthesis of 2-oxazoline monomers from carboxylic acids (**top**) and the cationic ring-opening polymerization of 2-oxazolines (**bottom**).

Poly(2-oxazoline)s can be synthesized from the cationic ring-opening polymerization of 2-oxazolines (Scheme 1). Their common structural motif is the amide bond of their side-chains; hence, poly(2-oxazoline)s may be referred to as pseudo-polyamides. The properties of these polymers can be adjusted by *in situ* and polymer analogous reactions [1,2,3]. In the last decade, crosslinking of these materials by (co)polymerizations with (at least) bifunctional 2-oxazoline monomers (*in situ* approach) [4,5] as well as the UV-induced crosslinking (often by “click” reactions during a polymer analogous approach) [6,7,8] have received renewed attention, benefiting in part from the increased research activities in the area of poly(2-oxazoline)s that coincides with the advent of microwave reactors in polymer scientists’ laboratories [9,10]. A dedicated focus is directed towards the application of crosslinked poly(2-oxazoline)s in the medicinal sector [11,12].

**Scheme 2:**
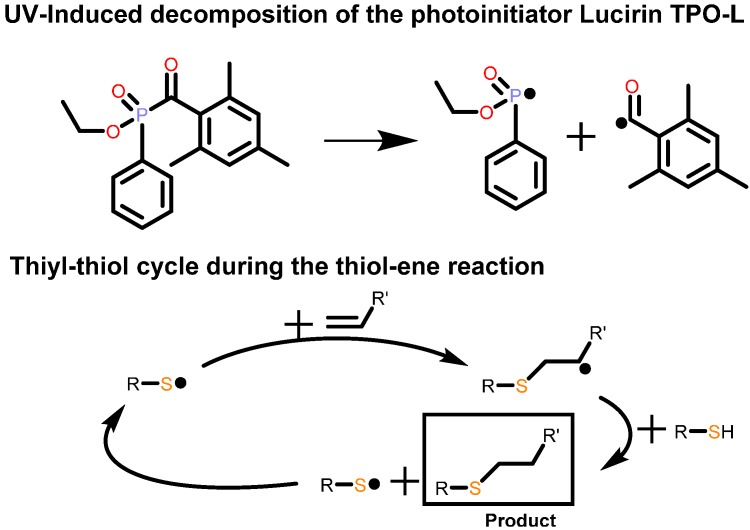
Schematic representations of the decomposition of the photoinitiator (**top**) and the thiol-thiyl cycle of the thiol-ene reaction (**bottom**).

UV-induced polymer analogous crosslinking of (co)poly(2-oxazoline)s by, e.g., the thiol-ene click reaction (Scheme 2), offers numerous advantages over the *in situ* crosslinking, despite the fact that an additional reaction step is required: The formulation containing the “ene” component (commonly a (co)poly(2-oxazoline) with olefinic functionalities in the side-chains), the “thiol” component (which, for crosslinking, must be an oligofunctional thiol), the UV-labile photoinitiator such as Irgacure TPO-L (2,4,6-trimethylbenzoylphenyl phosphinate; Scheme 2), and the solvent may be spin- or dropcast prior to the removal of the solvent, enabling the production of specimen with the envisaged geometry. As the thiol-ene reaction can be initiated by UV stimuli, the crosslinking reaction may proceed at room temperature, and polymer degradation and/or decomposition of temperature-sensitive parts of the network are kept at a minimum. Furthermore, spatial resolution of the crosslinking reaction can be achieved if the UV irradiation is applied through a geometric mask, yielding 2.5-dimensional polymer structures after dissolving the non-crosslinked parts of the polymer film. The term “2.5-dimensional” refers to the fact that a three-dimensional structure cannot be generated by this technique; the surface of the substrate is either covered by a photoresist (with identical height along the substrate) or not covered at all [13,14,15].

Notably, 2-oxazoline monomers with olefinic functionalities can be synthesized from the reaction of ethanol amine with fatty acids from renewable resources such as undec-10-enoic acid from castor oil [15]. This “green approach” towards the fabrication of polymer-based networks opens a plethora of novel application fields for this type of materials, including electronic devices such as pacemaker leads and high-voltage engineering devices such as “green” transformers.

From this background, the present study aimed at determining the dielectric characteristics of crosslinked (co)poly(2-oxazoline)s from renewable resources. This study was motivated in particular by the demand for novel, polymer-based insulator materials with low or no impact on the production environment and “switchable” crosslinking routines, e.g., crosslinking reactions that take place only after the application of stimuli such as UV irradiation. In contrast to thermally induced crosslinking that commonly starts immediately after the application of the polymer-based insulator formulation, the stimuli-triggered crosslinking renders more flexibility to production routines in general and to their schedule in particular. Furthermore, highly crosslinked polymer networks inherently offer advantages over their non-crosslinked analogues, such as higher rigidity/strength, insolubility, and the absence of a melting point. Consequently, a large field of (potential) applications opens for insulators based on polymer networks, comprising high-voltage outdoor applications [16,17]. Hence, three different poly(2-oxazoline)-based networks (with varying crosslinking degrees and composition of repetition units) were synthesized in this study in order to establish a first (fundamental) data set for the electronic properties of “green” (co)poly(2-oxazoline)-based networks. In particular, the permittivity, loss factor, and electrical conductivity were focused on, with special respect to the comparison with polymer classes such as polyamides, polyesters, and epoxy resins currently used as insulators in electronic applications (Table 1) [18].

**Table 1:** Density (at 20 °C), dielectric parameters (permittivity and loss factor, both at 20 °C and 50 Hz), and conductivity (20 °C, 50% r.h.) of polyamides, polyesters, and epoxy resins [18].

Parameter	Polymer		
	Polyamides	Polyesters	Epoxy Resins
Density (g·cm^−3^)	1.13–1.21	1.22–1.26	1.10–1.25
Permittivity	3.6–7	3–4.9	3.7–4.2
Loss factor	0.014–0.15	0.008–0.06	0.007–0.009
Conductivity (S·m^−1^)	10^−11^–10^−6^	10^−13^–10^−12^	10^−15^–10^−14^

## 2. Experimental Section

### 2.1. Materials

Unless indicated otherwise, all materials have been purchased from Sigma-Aldrich (Vienna, Austria). Irgacure TPO-L was purchased from ABCR (Karlsruhe, Germany). The 2-oxazoline monomers were prepared according to literature protocols [19,20]. Methyl tosylate was distilled prior to use, all other chemicals were used as received. The solvents acetonitrile and dichloromethane were purchased at Carl Roth (Vienna, Austria).

### 2.2. Instrumentation

^1^H-NMR and ^13^C-NMR spectra were acquired on a Bruker 300 MHz NMR spectrometer (Bruker BioSpin Corporation, Billerica, MA, USA) with a relaxation times of 4 s and 32 scans for ^1^H-NMR spectra, and a relaxation time of 2 s and 1024 scans for ^13^C-NMR spectra. The solvent signal of CDCl_3_ at 7.26 ppm for ^1^H-NMR spectra (77 ppm for ^13^C-NMR spectra) was used for referencing. All polymer syntheses were performed with a Biotage Initiator 8 microwave reactor (Biotage, Uppsala, Sweden) at a temperature of 140 °C. The microwave vials were dried prior to use at 80 °C for at least 1 h. FT-IR spectra were recorded on a Bruker Alpha FT-IR spectrometer (Bruker Optics Inc., Billerica, MA, USA) applying an ATR unit over a spectral range from 500 to 4000 cm^−1^. A background correction was performed prior to the measurements. For each sample, 32 scans were recorded. Size-exclusion chromatography (SEC) measurements were performed on a Shimadzu SEC system (Shimadzu Austria, Vienna, Austria) with a Shimadzu LC-20AD pump, a SIL-20ACHT sampler and an RID202A refractive index detector. A styrene-divinyl benzene copolymer network-based linear XL 5 µm column (PSS-SDV by Polymer Standards Service, Mainz, Germany) was used. As eluent, a mixture of chloroform/triethylamine/*iso*-propanol (94/4/2) was used at a flow rate of 1 mL/min. The results were referenced to polystyrene standards. For the production of test specimens, a Collin platen press P 200 PV (Dr. Collin GmbH, Ebersberg, Germany) was used. For the irradiation of the samples, a Novacure broadband UV-lamp by EFOS (EFOS, Mississauga, ON, Canada) was applied, and the samples were irradiated at 4500 mW·cm^−2^ from a distance of 10 cm. Conductivity measurements were performed in a three-terminal cell with a Keithley 617 electrometer (Tektronix, Eindhoven, The Netherlands). The cell was equipped with a protective serial resistor. The poling voltage was supplied via a Rogowski-profiled electrode made of aluminum. For the dielectric characterization, a Novocontrol Alpha-A dielectric analyzer (Novocontrol Technologies, Montabaur, Germany) was used applying a ZGS Alpha extension test interface as active cell. For temperature control, a Quatro cryosystem (Novocontrol Technologies, Montabaur, Germany) with an accuracy and stability of 0.1 and 0.01 K, respectively, was used. For the measurement of the dielectric properties, a sinoidal voltage of 3 V was applied to the samples.

### 2.3. Polymer Syntheses

All poly(2-oxazoline)s and copoly(2-oxazoline)s were synthesized from the corresponding monomers according to literature protocols [21].

#### 2.3.1. Poly(2-nonyl-2-oxazoline)_80_-*stat*-poly(2-dec-9′-enyl-2-oxazoline)_20_, pNonOx_80_-*stat*-pDc^=^Ox_20_

For the experiment, 81.2 mg of methyl tosylate were dissolved in 10 mL of dry dichloromethane in a 20 mL microwave vial. 1.860 g of 2-dec-9′-enyl-2-oxazoline and 6.865 g of 2-nonyl-2-oxazoline were added. Subsequently, the vial was sealed under inert conditions and the mixture heated at 140 °C for 2 h. The product was recovered as a white solid in a quantitative yield by removal of the solvent.

^1^H-NMR (20 °C, 300 MHz, CDCl_3_): δ (ppm) = 0.88 (246 H), 1.26 (1198 H), 1.59 (203 H), 2.42–2.33 (205 H), 3.44 (400 H), 4.91–5.01 (41 H), 5.73–5.87 (21 H).

^13^C-NMR (20 °C, 75 MHz, CDCl_3_): δ (ppm) = 14.0, 22.6, 25.2, 25.4, 28.9, 29.1, 29.3, 29.5, 31.9, 32.8, 33.0, 33.7, 43.1, 45.2, 114.1, 139.0, 173.2, 173.8.

FT-IR: ν(cm^−1^) = 2955, 2922, 2857, 1738, 1634, 1539, 1464, 1426, 1417, 1379, 1363, 1242, 1209, 1181, 1158, 1115, 988, 908, 775, 728, 681, 582.

GPC measurements: *M*_n_ = 9.7 kDa; *M*_w_ = 14.2 kDa; *M*_w_/*M*_n_ = 1.47.

#### 2.3.2. Poly(2-dec-9′-enyl-2-oxazoline)_100_, pDc^=^Ox_100_

Seventy-five milligrams of methyl tosylate were dissolved in 9 mL of dry dichloromethane in a 20 mL microwave vial. 8.450 g of 2-dec-9′-enyl-2-oxazoline were added. The vial was closed under inert conditions and the mixture was heated at 140 °C for 2 h. The product was recovered in a quantitative manner as a white solid after removal of the solvent under reduced pressure.

^1^H-NMR (20 °C, 300 MHz, CDCl_3_): δ (ppm) = 1.29 (1015 H), 1.59 (218 H), 2.02-2.04 (208 H), 2.32 (208 H), 3.44 (400 H), 4.91–5.01 (201 H), 5.73–5.87 (99 H).

^13^C-NMR (20 °C, 75 MHz, CDCl_3_): δ (ppm) = 25.2, 25.4, 25.7, 28.9, 29.1, 29.4, 29.5, 32.8, 32.9, 33.7, 43.4, 45.2, 114.1, 139.0, 173.1, 174.0.

FT-IR: ν(cm^−1^) = 3079, 2925, 2853, 1737, 1635, 1547, 1462, 1429, 1417, 1317, 1283, 1163, 989, 904, 771, 718, 637, 581.

GPC measurements: *M*_n_ = 7.2 kDa; *M*_w_ = 16.1 kDa; *M*_w_/*M*_n_ = 2.24.

#### 2.3.3. Preparation of the Test Specimens

Crosslinking of the (co)poly(2-oxazoline)s was performed analogously to UV-induced thiol-ene reactions described in the literature [13]. For the samples based on pNonOx_80_-*stat*-pDc^=^Ox_20_ (to be crosslinked with trimethylolpropane tris(3-mercaptopropionate) 3SH), 2.00 g of the polymer were dissolved in 25 g of dichloromethane, and 267 mg of trimethylolpropane tris(3-mercaptopropionate) as well as two drops of Irgacure TPO-L were added (ratio thiol:ene = 1:1). The mixture was stirred for 5 min, and the solvent was removed under reduced pressure (1.5 mbar) at 50 °C for 15 min. Two grams of the dry residue were transferred into a circular mold with a diameter of 50 mm and a height of 0.4 mm, which was situated between two steel plates. For a facilitated removal of the samples from the mold, a teflon foil was put between the mold and the plates. The stack was put into the platen press at 110 °C and 25 bar. The temperature was increased to 125 °C, and heating was continued for 2.5 min under vacuum. Finally, the samples were cooled down to 35 °C and removed from the mold. For the crosslinking of the polymer, the samples were irradiated with UV-light. For the samples based on pDc^=^Ox_100_ (to be crosslinked with trimethylolpropane tris(3-mercaptopropionate) 3SH), 2.05 g of the polymer were dissolved in 10 mL of dichloromethane. Prior to stirring for 5 min, 1.28 g of trimethylolpropane tris(3-mercaptopropionate) and 15 mg of Irgacure TPO-L were added (ratio thiol:ene = 1:1). The solvent was removed under reduced pressure (1.5 mbar) for 15 min. Into a circular mold with a diameter of 50 mm and a height of 0.4 mm, 2.36 g of the solid mixture was placed. The mold was situated between two steel plates, which were covered with Teflon foil for facilitated removal of the samples. The steel plates were put into a preheated platen press at a temperature of 110 °C and 25 bar. After increasing the temperature to 135 °C, the samples were pressed for 10 min under vacuum, subsequently cooled and removed from the mold. Subsequently, the samples were irradiated with UV-light. For the samples based on pDc^=^Ox_100_ (to be crosslinked with 1,3-dimercaptobenzene 2SH), 1.055 g of the polymer were dissolved in 5 mL of dichloromethane and 0.355 g of 1,3-dimercaptobenzene as well as 20 mg of Irgacure TPO-L were added. The solutions were poured into a cast with 50 mm diameter. The solvent was evaporated, and the samples were illuminated by UV light. Subsequently, the samples were dried in a vacuum oven at 40 °C for 48 h.

## 3. Results and Discussion

### 3.1. Choice of Crosslinked (co)poly(2-oxazoline)s

**Scheme 3:**
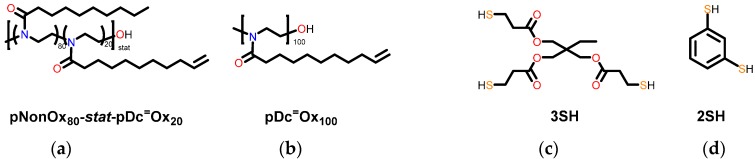
Reactants for the thiol-ene reactions used in this study: (co)poly(2-oxazoline)s as ene components (**a** and **b**) and di- and trimercapto compounds as thiol components (**c** and **d**).

Both types of 2-oxazoline monomers, namely 2-nonyl-2-oxazoline NonOx and 2-dec-9′enyl-2-oxazoline Dc^=^Ox, could be synthesized from the titanium alkoxide-catalyzed reaction of ethanol amine [19,20] with decanoic acid (from renewable resources such as coconut oil [22]) and undec-10-enoic acid (from renewable resources such as castor oil [23]) (Scheme 1). Their cationic ring-opening polymerization and copolymerization (Scheme 1), respectively, can be initiated by initiators such as methyl tosylate and be conveniently performed in microwave reactors [10,24] within reasonably short reaction times of 2 h. For this study, with respect to subsequent thiol-ene crosslinking (see below), a homopolymer of Dc^=^Ox and a copolymer of NonOx and Dc^=^Ox were synthesized as ene components (Scheme 3). The corresponding polymers pNonOx_80_-*stat*-pDc^=^Ox_20_ and pDc^=^Ox_100_ can be crosslinked in UV-induced polymer analogous thiol-ene reactions employing the unsaturated side-chain functionalities of the pDc^=^Ox repetition units. This approach inherently bears the advantage that the thiol and the (co)poly(2-oxazoline) (as well as the photoinitiator) can be cast into the targeted shape from, e.g., solution-based processes, and be crosslinked in that shape, enabling the straightforward production of test specimen with dedicated geometry. For crosslinking, at least bisfunctional thiols and “enes” (polymers with at least two repetition units of pDc^=^Ox) must be employed.

In order to investigate the effect of crosslinking on the electrical conductivity and the permittivity, pNonOx_80_-*stat*-pDc^=^Ox_20_ and pDc^=^Ox_100_ were crosslinked with a trisfunctional thiol 3SH (trimethylolpropane tris(3-mercaptopropionate)); in addition, pDc^=^Ox_100_ was also crosslinked with a bisfunctional thiol 2SH (1,3-dimercaptobenzene) (Scheme 3). All reaction mixtures were calculated such that a thiol:ene ratio = 1:1 was maintained, assuming quantitative reactions [25]. Notably, a ratio of 80:20 = 4:1 of “crosslinkable” *vs.* “non-crosslinkable” repetition units (as in pNonOx_80_-*stat*-pDc^=^Ox_20_) had been found to yield insoluble polymer networks in a previous study [13] and was therefore included in these investigations as well. Prior to electrical characterization, all samples were dried thoroughly in order to exclude the effect of moisture on the polarization.

The maximum degree of crosslinking can be calculated from the averaged molecular weight per polymer analogously formed bond (*M*_BOND_): The (maximum) number of novel (crosslinking) bonds per polymer chain is equal to the number of its Dc^=^Ox repetition units; for the formation of these bonds, one polymer chain and the corresponding number of thiols are required (maintaining the ratio thiol:ene = 1:1) (Equation (1)).

(1) $${\text{M}}_{\text{BOND}}=\frac{\text{M}\left[\left(\text{co}\right)\text{poly}\left(2-\text{oxazoline}\right)\right]+\left\{\text{M}\left[\text{thiol}\right]\cdot \frac{{\text{number of pDc}}^{=}\text{Ox repetition units}}{\text{functionality of the thiol}}\right\}}{{\text{number of pDc}}^{=}\text{Ox repetition units}}$$

From the as-calculated values of *M*_BOND_ for the three types of polymer networks investigated in this study (Table 1), the (experimentally determined) density of the crosslinked (co)poly(2-oxazoline)s, and the functionality of the thiol, the “knot density” ρ_KNOT_ can be calculated correspondingly (Equation (2), Table 2).

(2) $$\rho_{\text{KNOT}}=\frac{\rho }{M_{\text{BOND}}}\cdot (\text{functionality of the thiol})$$

**Table 2:** Molecular weights per polymer analogously formed bond in the three (co)poly(2-oxazoline)-based networks investigated in this study.

Type of Network	*M*_BOND_ (g·moL^−1^)	ρ (g·cm^3^) at 25 °C	ρ_KNOT_ (mmol·cm^−3^)
pNonOx_80_-*stat*-pDc^=^Ox_20_ (CL:3SH)	1663	0.992	1.8
pDc^=^Ox_100_ (CL:2SH)	281	1.116	8.0
pDc^=^Ox_100_ (CL:3SH)	342	1.184	10.5

### 3.2. Relative Permittivity

The relative permittivity quantifies the ratio of the capacitance of a capacitor filled with a dielectric material to that of the same capacitor in vacuum. The relative permittivity increases with the polarizability of the material, in particular with its ability to store charges [26]. As the material’s polarization does not react instantaneously to external stimuli, the response to an applied frequency commonly occurs with a phase shift, and the relative permittivity is best described as a complex function (Equation (3)), in which ε_R_′ is the real part of the permittivity (quantifying the capability of the material to store energy), and ε_R_′′ is the imaginary part of the permittivity (quantifying the loss of energy from the material) [26].

(3) ε_R_ = ε′_R_ − *i*·ε′′_R_

**Figure 1:**
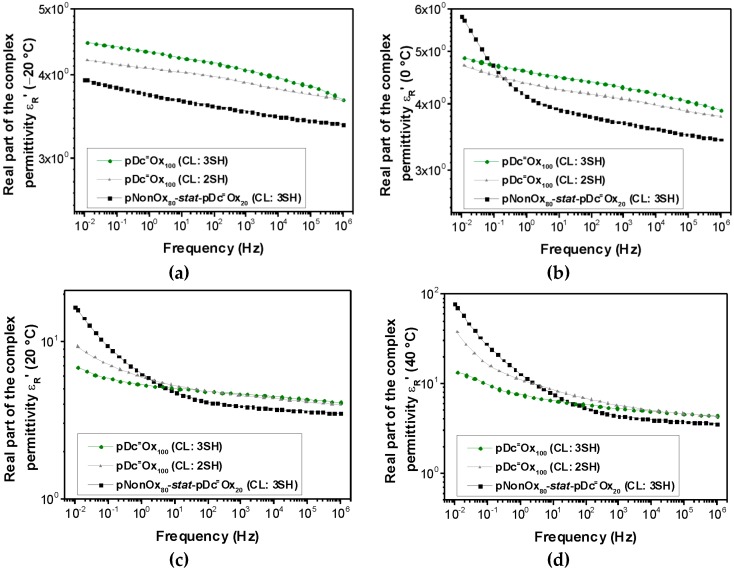
Frequency-dependent real part of the complex permittivity of pNonOx_80_-*stat*-pDc^=^Ox_20_ (CL:3SH), pDc^=^Ox_100_ (CL:2SH), and pDc^=^Ox_100_ (CL:3SH) at −20 °C (**a**), 0 °C (**b**), 20 °C (**c**) and 40 °C (**d**).

**Figure 2:**
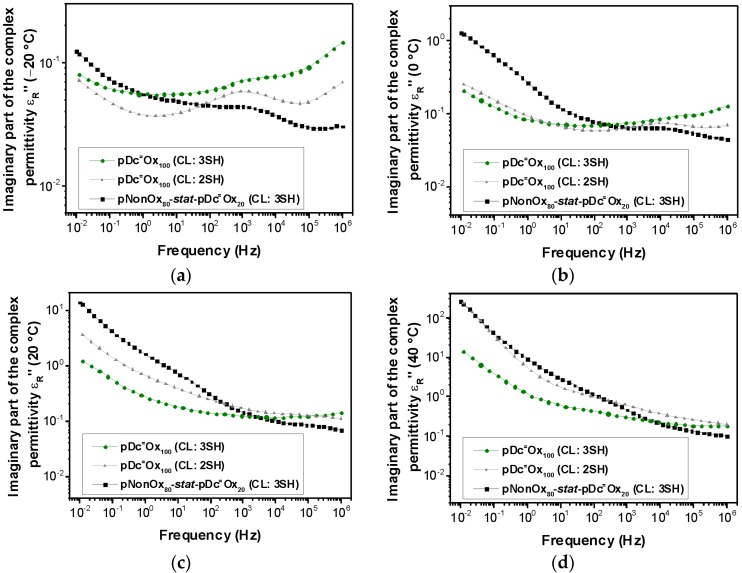
Frequency-dependent imaginary part of the complex permittivity of pNonOx_80_-*stat*-pDc^=^Ox_20_ (CL:3SH), pDc^=^Ox_100_ (CL:2SH), and pDc^=^Ox_100_ (CL:3SH) at −20 °C (**a**), 0 °C (**b**), 20 °C (**c**) and 40 °C (**d**).

Both the real and the imaginary part of the complex permittivity, are commonly dependent on the temperature and the frequency. The relative permittivity of the three types of (co)poly(2-oxazoline)-based networks has been measured at four different temperatures (−20, 0, 20, and 40 °C) and frequencies ranging from 10^−2^ to 10^6^ Hz (Figure 1 to Figure 2). The permittivity at 50 Hz (the industrial standard; Table 3) of all network types increases with the temperature. At 20 °C, a permittivity from 4.29–4.97 can be observed, which is in the expected range for polymers with low to medium polarity such as polyamides, polyesters, or epoxy resins (Table 1).

**Table 3:** Permittivity of the poly(2-oxazoline)-based networks at 50 Hz.

Polymer Network	Permittivity			
	*T* = −20 °C	*T* = 0 °C	*T* = 20 °C	*T* = 40 °C
pNonOx_80_-*stat*-pDc^=^Ox_20_ (CL:3SH)	3.62	3.81	4.29	5.81
pDc^=^Ox_100_ (CL:2SH)	4.00	4.19	4.97	7.28
pDc^=^Ox_100_ (CL:3SH)	4.19	4.42	4.97	6.04

Notably, while the magnitude of the real part of the complex permittivity at 50 Hz is not altered significantly within the temperature range from −20 to 40 °C (maximum increase in the case of pDc^=^Ox_100_ (CL:2SH) by a factor of 1.8), the imaginary part of the complex permittivity increases significantly (details of these phenomena have been summarized hereinafter; Section 3.3.). A more detailed investigation of the frequency-dependent real part of the complex permittivity (Figure 1) reveals that, at −20 °C, an overall linear correlation between the (logarithmic) frequency and the (logarithmic) real part of the complex permittivity can be observed. At 0 °C, this linear correlation along the whole range of frequencies can be only observed for pDc^=^Ox_100_ (CL:2SH) and pDc^=^Ox_100_ (CL:3SH); for pNonOx_80_-*stat*-pDc^=^Ox_20_ (CL:3SH), deviations from the linear correlation occur for frequencies lower than 1 Hz. At 20 and 40 °C, all types of networks show significant deviation from the linear correlation for frequencies lower than 1–10 Hz (20 °C) and 10–100 Hz (40 °C). In summary, the occurrence of this phenomenon depends on the temperature and the type of polymer network (pNonOx_80_-*stat*-pDc^=^Ox_20_ (CL:3SH) with a comparably low network density *vs.* pDc^=^Ox_100_ (CL:2SH) and pDc^=^Ox_100_ (CL:3SH) with a high degree of crosslinking; Table 2).

Hence, the relative permittivity and, as its origin, the polarizability can be significantly influenced by the temperature and network density. While a significant influence of the temperature on the permittivity and polarizability was expected, the influence on the polarizability by the network density requires a detailed inspection: electronic polarization (caused by frequencies in the range of 10^15^ Hz), atomic polarization (caused by frequencies in the range of 10^13^ Hz), and orientation polarization (caused by frequencies in the range of 10^9^ Hz) can be excluded to cause these phenomena [26].

At the lower range of frequencies measured in this study (lower than 1–10 Hz at 20 °C and 10–100 Hz at 40 °C, respectively), only interfacial polarization with typical frequencies of 10^−5^ to 10^2^ Hz can be assumed to cause the polarization effects. For frequencies higher than 10^2^ Hz, interfacial polarization decreases significantly, which is also observed in this study for that range of frequencies (Figure 1 to Figure 2). As a rule of thumb, interfacial polarization occurs in all polymers with structural inhomogenities. Notably, water contaminations may also affect polarization in that range of frequencies; this origin of low-frequency polarization, however, can be excluded due to extended drying procedures prior to the measurements. The frequency at which interfacial polarization starts to occur increases with the temperature and the “degree of inhomogeneity” [26]. Due to the fact the observed phenomena are most pronounced in the case of pNonOx_80_-*stat*-pDc^=^Ox_20_ (CL:3SH), both the composition of the (co)poly(2-oxazoline)s and the network density seem to influence this polarization.

### 3.3. Loss Factor

While ε_R_′ (Figure 1) quantifies the material’s capability to store energy and, hence, is indicative of the material’s polarizability (see hereinabove), the imaginary part ε_R_′′ (Figure 2) quantifies the losses of energy. Commonly, the conductivity of a material is classified based on the loss factor tan δ, which is defined as the ratio of the imaginary part of the complex permittivity and the real part of the complex permittivity (Equation (4)) [26].

(4) $$\tan\delta =\frac{\varepsilon_{R}\prime \prime }{\varepsilon_{R}\prime }$$

**Figure 3:**
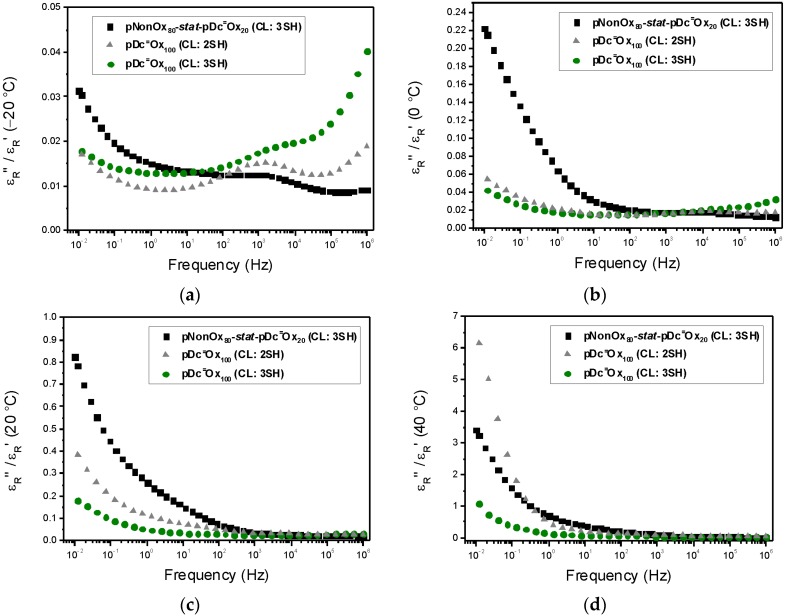
Frequency-dependent loss factors of pNonOx_80_-*stat*-pDc^=^Ox_20_ (CL:3SH), pDc^=^Ox_100_ (CL:2SH), and pDc^=^Ox_100_ (CL:3SH) at −20 °C (**a**), 0 °C (**b**), 20 °C (**c**) and 40 °C (**d**).

An ideal dielectric would show no loss of energy (tanδ = 0), while a perfect conductor would not store any energy (tanδ = ∞), As a rule of thumb, materials with tanδ << 1 can be considered as good dielectrics and, consequently, as poor conductors. A detailed investigation of the loss factors (Figure 3) reveals that, at −20 °C, all poly(2-oxazoline)-based networks are poor conductors (independent of the frequency). At 0 °C, only pDc^=^Ox_100_ (CL:2SH) and pDc^=^Ox_100_ (CL:3SH) may be considered good dielectrics over the whole range of frequencies; at frequencies lower than 1 Hz, pNonOx_80_-*stat*-pDc^=^Ox_20_ (CL:3SH) starts to become a conducting material. At 20 and 40 °C, only pDc^=^Ox_100_ (CL:3SH) acts as a good dielectric (for frequencies higher than 1 Hz); pNonOx_80_-*stat*-pDc^=^Ox_20_ (CL:3SH) and pDc^=^Ox_100_ (CL:2SH), on the other hand, may be classified as lossy conducting materials for frequencies lower than 1 Hz (at 40 °C). In summary, the trends observed for the polarizability of the poly(2-oxazoline)-based networks (Section 3.2.) are well reproduced with the loss factor; the interfacial polarization, hence, suffices to explain the dielectric properties of these network in the range of frequencies from 10^−2^ to 10^6^ Hz. The standardized values of the loss factor (at 20 °C and 50 Hz; Table 4) emphasize the classification of poly(2-oxazoline)s as (pseudo-)polyamides. Both polyesters as well as epoxy resins typically have lower loss factors (Table 1).

**Table 4:** Loss factors of the poly(2-oxazoline)-based networks at 50 Hz.

Polymer Network	Loss Factor			
	*T* = −20 °C	*T* = 0 °C	*T* = 20 °C	*T* = 40 °C
pNonOx_80_-*stat*-pDc^=^Ox_20_ (CL:3SH)	0.014	0.024	0.093	0.262
pDc^=^Ox_100_ (CL: 2SH)	0.010	0.014	0.054	0.159
pDc^=^Ox_100_ (CL:3SH)	0.014	0.016	0.030	0.076

### 3.4. Electrical Conductivity

**Figure 4:**
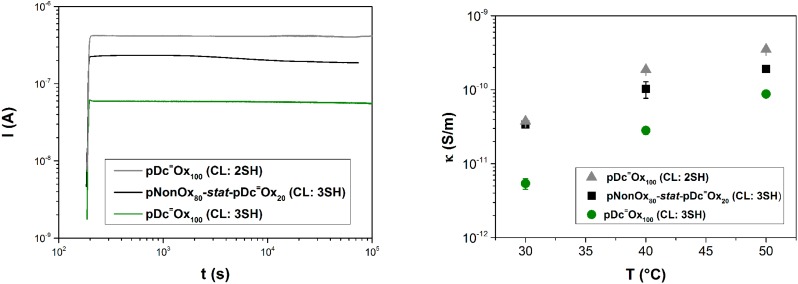
Measurements of the electrical conductivity of the poly(2-oxazoline)-based networks. **(Left)**: Time-dependent set-up of constant current conditions; (**Right**): Electrical conductivities for the polymer networks at 30, 40, and 50 °C.

In order to thoroughly characterize these poly(2-oxazoline)-based networks, also their electrical conductivity was measured. These measurements were performed on (thoroughly dried) polymer disks at 30, 40, and 50 °C. Constant current conditions (quasi steady-state conditions) were achieved for all samples in the range of days due to slow polarization and conduction procedures, while, on the other hand, pseudo steady-state conditions were achieved in the range of hours (Figure 4; left). As expected, the electric conductivity was found to correlate positively with the temperature (Figure 4; right). pDc^=^Ox_100_ (CL:3SH) showed the lowest electrical conductivity of all samples in the range from 5 × 10^−12^ to 10^−10^ S/m. In total, a range of electrical conductivities in the range from 5 × 10^−12^ to 8 × 10^−9^ S/m was covered by the poly(2-oxazoline)-based networks. However, these values might be (slightly) lowered if the time range of multiple days or even weeks was allowed for determining the steady-state conditions. For the range of electrical conductivities measured for the poly(2-oxazoline) networks, again their similarity with polyamides becomes discernible; polyesters and epoxy resins commonly have lower conductivities (Table 1). The range of conductivities classifies these materials as medium insulators (semiconducting and conducting polymers have conductivities higher than 10^−9^ S/m [27]).

## 4. Conclusions

Two types of (co)poly(2-oxazoline)s, namely pNonOx_80_-*stat*-pDc^=^Ox_20_ and pDc^=^Ox_100_, were prepared by the cationic ring-opening polymerization of 2-oxazoline monomers, which were synthesized by the reaction of ethanol amine with fatty acids from renewable resources. These polymers could be crosslinked by the UV-induced thiol-ene reaction with a di- or a trithiol, respectively. The corresponding polymer networks exhibited permittivities in the range of 4.29–4.79 and loss factors in the range of 0.030–0.093 (20 °C, 50 Hz). Interfacial polarization within the materials was found to alter the real part of their permittivity and their loss factor from frequencies lower than 100 Hz. The electrical conductivity of these networks is in the range from 5 × 10^−12^ to 8 × 10^−9^ S/m, which renders these materials medium insulators. These first data of the dielectric and electronic properties of the investigated (co)poly(2-oxazoline)-based networks, which may be described as pseudo-peptides, show a high degree of similarity to polyamides, which qualifies the (co)poly(2-oxazoline)s as potential “green” alternatives to polyamides as insulators in electronic applications.
